# The conceptualization of terms: ‘Mood’ and ‘affect’ in academic trainees of mental health

**DOI:** 10.4103/0019-5545.58295

**Published:** 2009

**Authors:** Narayana Manjunatha, Christoday Raja Jayant Khess, Dushad Ram

**Affiliations:** Department of Psychiatry, National Institute of Mental Health and Neuro Sciences (NIMHANS), Bangalore - 560 029, India; 1 Central Institute of Psychiatry, Kanke, Ranchi, India; 2 JSS Medical College, Mysore, Karnataka, India

**Keywords:** Mood, affect, conceptualization, mental health trainees

## Abstract

**Background:**

The management of psychiatric disorders should ideally be carried out by a multidisciplinary team that consists of mental health professionals from different disciplines. All mental health professionals are expected to learn similar basic clinical skills during their training, despite the difference in their graduation.

**Objective:**

To compare the conceptualization of the terms ‘mood’ and ‘affect’ in all academic trainees of mental health in the Central Institute of Psychiatry (CIP), Ranchi, India.

**Materials and Methods:**

The ‘modified mood and affect questionnaire’ administered to all mental health trainees of CIP, Ranchi, India, in this study. The participants were requested to mark one response (either ‘true’, ‘false’ or ‘not sure’) for each item. The completed questionnaire was collected on the spot.

**Results:**

The statistical analysis was done for the data from psychiatric residents and trainees of clinical psychology. The statistical differences were observed between these two groups in response to the items-‘Mood is the moment to moment emotional tone’ and items of ‘sign/symptom dimension’.

**Conclusions:**

The observed statistical difference in items could be the reflection of the differences in the description of ‘mood’ and ‘affect’ in textbooks of psychopathology, as well as, the difference in their graduation. The trainees of clinical psychology may be benefitted with more exposure in medical knowledge during their training.

## INTRODUCTION

Ideally, the management of mental and behavioral disorders is carried out by a team of multidisciplinary mental health professionals consisting of psychiatrists, clinical psychologists, psychiatric social workers, psychiatric nurses, and others. This may not be true in psychiatric practices in India. However, a majority of the teaching mental health institutes in India have multidisciplinary team consisting of the above-mentioned professionals, with the aim to improve the clinical services, academic as well as research activities. Every mental health professional, during his/her training, irrespective of disciplines, is expected to learn similar clinical skills and deliver clinical services. All the trainees of mental health, irrespective of the discipline, are expected to learn similar basic skills of Mental Status Examination (MSE) during their training. Hence, it is interesting to compare some of the core skills of MSE in the trainees of different mental health disciplines.

The assessment of ‘mood’ and ‘affect’ is a vital part of MSE and is the cornerstone in the clinical assessment of mental and behavioral disorders.[1] The changes in mood and affect are paramount in taking clinical decisions during the management of different psychiatric disorders.[2] The descriptions of the terms ‘mood’ and ‘affect’ are adequately covered elsewhere.[2] However, the descriptions of these terms may be understood differently by trainees of different disciplines of mental health professions. Therefore, it is essential to understand the differences in the conceptualization of the terms ‘mood’ and ‘affect’ among trainees of different disciplines of mental health. If the differences exist, further efforts may be carried out to reduce the differences and improve inter-rater reliability, which in turn improves the understanding of basic skills and ultimately the delivery of clinical services.

This study aims at comparing the conceptualizations of the terms ‘mood’ and ‘affect’ in all disciplines of mental health trainees in our institute. Authors hypothesized that there would be no differences in the understanding of the terms ‘mood’ and ‘affect’, as they were all undergoing similar training in the same institute.

## MATERIALS AND METHODS

The original mood and affect questionnaire of Serby[3] that was modified to suit for this study has already been dealt elsewhere.[2] The final ‘modified mood and affect questionnaire’[2] was prepared after a discussion with teachers of all disciplines, to improve the validity.

This study was conducted in the Central Institute of Psychiatry (CIP), Ranchi, India, which is a premier postgraduate mental health institute in Eastern India, imparting training in psychiatry, clinical psychology, psychiatric social work, and psychiatric nursing, with the focus on multidisciplinary approach. The CIP has both in- and out-patient services along with more than 600 inpatient beds. The CIP also has an output of at least fifteen psychiatrists, fifteen clinical psychologists, six psychiatric social workers, and six psychiatric nurses every year. This study was also approved by the Institutional Review Board of CIP, Ranchi, India.

The final ‘modified mood and affect questionnaire’[2] was administered to all the trainees of the different disciplines of our institute [psychiatric residents (PR) of both DPM and MD, trainees of clinical psychology (CP), including M.Phil and PhD, trainees of psychiatric social work (M.Phil), and trainees of psychiatric nursing (DPN)] and they were invited for participation in this study. After their informed consent, all the trainees were requested to mark one response (one of either ‘true’ or ‘false’, or ‘not sure’) for each item. The completed questionnaire was collected back on the spot after the stipulated time. Forty-one out of 43 PR, 38 of 40 CP, seven psychiatric social work, and six psychiatric nurse trainees participated in this study. The statistical analysis was done for responses of PR (N = 43) and CP (N = 38) only. The basic sociodemographic data of the participants (except their names, to maintain the anonymity) along with their disciplines and duration of training in mental health, were collected at the time of this study.

## RESULTS

The mean ages of PRs and CPs were 29.09 (SD-3.09) and 26.28 (SD-2.05) years, respectively. The means of the duration of training of PRs and CPs in mental health at the time of this study were 25.80 (SD-21.46) and 27.58 (SD-20.16) months, respectively. The result of the study is summarized in Table 1. A statistical significant difference was observed in the response to items, — ‘Mood is the moment to moment emotional tone’ and items of ‘sign/symptom dimension’. There was no statistical difference observed in items of sustained/momentary dimension, subjective/objective dichotomy, external/internal dimension, or using quotation mark/in the patient verbatim.

**Table 1:** The item-wise comparison of the responses of psychiatric residents and clinical psychologys in the questionnaire

No	items	PR (N-41)			CP (N-38)			χ^2^ test
				
		True (%)	False (%)	Not sure (%)	True (%)	False (%)	Not sure (%)	*P* value
1	Mood is the pervasive and sustained emotional tone	92.7	7.3	0	81.6	18.4	0	NS
2	Affect is the pervasive and sustained emotional tone	4.9	95.1	0	10.5	84.2	5.3	NS
3	Mood is the moment to moment emotional tone	12.2	87.8	0	26.3	65.8	7.9	.038
4	Affect is the moment to moment emotional tone	85.4	14.6	0	76.3	15.8	7.9	NS
5	Mood is the subjective description of emotional tone	68.3	22.0	9.8	65.8	28.9	5.3	NS
6	Affect is the subjective description of emotional tone	43.9	53.7	2.4	47.8	47.8	5.3	NS
7	Mood is the objectively observed emotional tone	19.5	73.2	7.3	34.2	60.5	5.3	NS
8	Affect is the objectively observed emotional tone	85.4	12.2	2.4	78.9	18.4	2.6	NS
9	Mood is internal	87.8	9.8	2.4	76.3	13.2	10.5	NS
10	Affect is internal*	29.3	63.4	7.3	39.5	50.0	7.9	NS
11	Mood is external*	14.6	75.6	9.8	13.2	68.4	15.8	NS
12	Affect is external	70.7	19.5	9.8	55.3	28.9	15.8	NS
13	Mood should be recorded in quotation mark/in the patient verbatim	48.8	43.9	7.3	36.8	52.6	10.5	NS
14	Affect should be recorded in quotation mark/in the patient verbatim	65.9	31.7	2.4	68.4	26.3	5.3	NS
15	Mood is a symptom	78.0	14.6	7.3	50.0	36.8	13.2	.032
16	Affect is a symptom	19.5	75.6	4.9	44.7	42.1	13.2	.010
17	Mood is a sign*	17.1	75.6	7.3	42.1	36.8	18.4	.006
18	Affect is a sign*	85.4	9.8	4.9	65.8	15.8	15.8	NS
19	Do you get adequate information about mood and affect in literature/text books	36.6	43.9	19.5	57.9	28.9	13.2	NS

* One trainee of clinical psychology not marked; *P* value < 05; NS = Non-significant; χ^2^ test = Chi-square test

## DISCUSSION

There are recent increases in literature on mood and affect.[4] The mood and affect sections of MSE were taken up in this study, as the changes in mood and affect are paramount in taking clinical decisions in the management of different psychiatric disorders. On account of the non-suitability of statistical tests in less number of responders in other disciplines, a statistical analysis was carried out only for responses of psychiatric residents (PRs) and trainees of clinical psychology (CPs), despite data being collected from all mental health trainees of the institute.

Authors observed the statistical differences between PRs and CPs in the items No. 15; 16, and 17 of Table 1, which could be the reflection of trends of trainees in the reading of both European and American textbooks, as well as the differences in their graduation background (will be discussed in detail a little later in the text).

About 88% of PRs and 66% of CPs have not considered mood as a moment-to-moment emotional tone [Item No. 3 in Table 1]. This may be the reflection of differences in two popular psychopathology books available in India to describe to terms ‘mood’ and ‘affect’ at the time of this study [Fish's psychopathology (Hamilton, 1984) and SIMS (2003)]. In Fish's clinical psychopathology,[5] mood and affect are described as follows: Strictly speaking, mood is the emotional state prevailing at any given time or, as Deese (p. 70) puts it, “the dominant hedonic tone of the moment”. However, mood is often used by psychiatrists for an emotional state that usually lasts for some time, and which colors the total experience of the subject, or in other words, ‘a mood state’. Thus, while an emotion is a short-lived response, a mood state is a lasting disposition, either reactive or endogenous, to react to events with a certain kind of emotion. On the other hand, ‘affects are waves of emotion in which there is a sudden exacerbation of emotion, usually as a response to some event’. However, in the next line, ‘strictly speaking, mood is the emotional state prevailing at any given time or dominant hedonic tone of moment’. One would conclude from the above italic sentences of Fish that affect is a sudden exacerbation of emotion, and mood is also the emotional state prevailing at any given time, in other words, both mood and affect are short-term emotional tone (However, these confusing lines are deleted in the new edition of Fish's clinical psychopathology). In other texts, for example, Sims[6] as well as glossary of DSM- IV TR.[7] there is a difference in these descriptions (described in detail a little later in the text). According to Sims,[6] Karl Jaspers described the terms mood and affect as follows: Affect is a complex but momentary emotional perturbation. Mood is a more prolonged emotional state, which influences all aspects of the mental state. It is clear from the above lines of Sims[6] that ‘affect is momentary, while mood is prolonged emotion’. The glossary of DSM-IV TR[7] defines the terms ‘mood’ and ‘affect’ in the line of Sims[6] as follows: ‘Affect is a pattern of observable behaviors that is an expression of subjectively experienced feeling state (emotion)’, whereas, ‘mood is a pervasive and sustained emotion that colors the perception of the world’. In other words, *mood* refers to a more pervasive and sustained emotional ‘climate’, whereas, *affect* refers to more fluctuating changes in the emotional ‘weather’. The glossary of DSM-IV TR[7] makes it clear that *‘affect is momentary (like weather)*, *while*, *mood is a prolonged emotion (like climate)’*. Authors feel that the differences in these popular texts of psychopathology may be attributed to the differences in conceptualizations among the German and the Anglo-American psychopathologists. Unfortunately, however, it is causing more confusion than clarity of the conceptualization of the terms ‘mood’ and ‘affect’.

The statistical differences observed in ‘sign/symptom dimension’ (items No. 15, 16, and 17) may be a reflection of their graduation training in India. During their ‘Bachelor of Medicine and Bachelor of Surgery’ (MBBS) graduation in India, the PRs are well exposed to the ‘sign/symptom dimension’ (pre-requisite qualification in India to enter post-graduation in psychiatry is MBBS), whereas, it is not so in the case of CPs in India (CPs come from non-clinical graduation like BA/BSc). This may suggest that CPs may be benefited with exposure to more medical terminology during their postgraduate mental health training, to reduce the above-mentioned differences.

There was no statistical difference in any other item in the questionnaire. This may be explained by the habit of reading similar texts of psychiatry (Comprehensive Textbook of Psychiatry and Oxford Textbook of Psychiatry) during their respective mental health post-graduate training in the same institute. However, a majority of PRs and CPs (approximately 44% of PR and 29% of CPs in item No. 19) felt that they were getting inadequate information about ‘mood’ and ‘affect’ in literatures/textbooks, which suggested that further convergent literatures were required in the concepts of mood/affect. Future studies may focus on other sections of MSE in multi-site international studies, with a higher number of participants.

## CONCLUSIONS

There are no significant differences in the responses of psychiatric and clinical psychology trainees in the questionnaire, except in the item ‘mood is the moment-to-moment emotional tone’ and in the item ‘sign/symptom dimension’, which could be the reflection of the difference in the description of ‘mood’ and ‘affect’ in textbooks of psychopathology, as well as, the difference in their graduation background. The trainees of clinical psychologists may get benefited from more exposure to medical terminology during their first clinical mental health training in India.
